# A 6.7 μW Low-Noise, Compact PLL with an Input MEMS-Based Reference Oscillator Featuring a High-Resolution Dead/Blind Zone-Free PFD

**DOI:** 10.3390/s24247963

**Published:** 2024-12-13

**Authors:** Ahmed Kira, Mohannad Y. Elsayed, Karim Allidina, Vamsy P. Chodavarapu, Mourad N. El-Gamal

**Affiliations:** 1 Department of Electrical and Computer Engineering, McGill University, Montreal, QC H3A 0G4, Canada; mourad.el-gamal@mcgill.ca; 2 MEMS Vision International Inc., Montreal, QC H4P 2R9, Canada; mohannad.elsayed@mems-vision.com (M.Y.E.); karim.allidina@mems-vision.com (K.A.); 3 Department of Electrical and Computer Engineering, University of Dayton, Dayton, OH 45469, USA; vchodavarapu1@udayton.edu

**Keywords:** frequency synthesizer, high resolution, low noise, microelectromechanical system (MEMS), oscillator, phase-frequency detector (PFD), phase-locked loop (PLL), time difference, timing, ultra-low power

## Abstract

This article reports a 110.2 MHz ultra-low-power phase-locked loop (PLL) for MEMS timing/frequency reference oscillator applications. It utilizes a 6.89 MHz MEMS-based oscillator as an input reference. An ultra-low-power, high-resolution phase-frequency detector (PFD) is utilized to achieve low-noise performance. Eliminating the reset feedback path used in conventional PFDs leads to dead/blind zone-free phase characteristics, which are crucial for low-noise applications within a wide operating frequency range. The PFD operates up to 2.5 GHz and achieves a linear resolution of 100 ps input time difference ($\Delta t_{in}$), without the need for any additional calibration circuits. The linearity of the proposed PFD is tested over a phase difference corresponding to aa $\Delta t_{in}$ ranging from 100 ps to 50 ns. At a 1 V supply voltage, it shows an error of <±1.6% with a resolution of 100 ps and a frequency-normalized power consumption ($P_{n}$) of 0.106 pW/Hz. The PLL is designed and fabricated using a TSMC 65 nm CMOS process instrument and interfaced with the MEMS-based oscillator. The system reports phase noises of −106.21 dBc/Hz and −135.36 dBc/Hz at 1 kHz and 1 MHz offsets, respectively. It consumes 6.709 μW at a 1 V supply and occupies an active CMOS area of 0.1095 mm^2^.

## 1. Introduction

Timing and frequency reference oscillators are ubiquitous in almost all electronic systems. Modern emerging applications like the Internet of Things (IoT), implantable medical devices, smart watches, and mobile devices are continuously demanding higher performance systems, imposing stringent requirements in terms of more compact integration, lower power consumption (battery life), better quality, and lower cost [1,2,3,4].

Recently, high-quality factor (*Q*) MEMS-based oscillators have become of great interest in replacing traditional quartz-based oscillators for precision timing reference and frequency synthesis [5]. MEMS resonators are orders of magnitude smaller than quartz crystals, which enables system miniaturization through a significant reduction in the overall system footprint while maintaining high performance levels [6,7,8,9,10,11]. For a MEMS-based oscillator, a phase-locked loop (PLL) plays a key role in either providing frequency correction of the fabrication process and temperature variations [12,13,14,15,16] or synthesizing different output frequencies for different applications [17,18,19]. It is a pivotal block that enables an efficient and a complete implementation of a MEMS oscillator.

**Figure 1:**
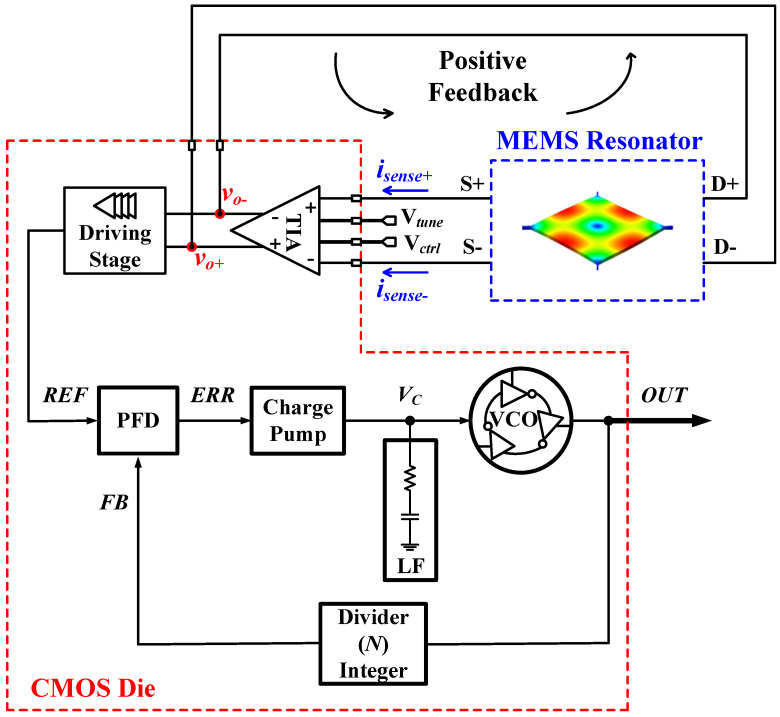
The PLL system with its MEMS-based input reference oscillator.

This paper reports the implementation of a MEMS-based frequency synthesizer. The resonator and the oscillator were discussed in detail in [20,21], respectively. This paper focuses on the PLL. Figure 1 shows an overall block diagram of the system, in which the MEMS resonator is wire-bonded to the CMOS die that contains the oscillator-sustaining amplifier circuit and the integer-N synthesizer. The oscillator output provides a 6.89 MHz input reference frequency to the integer-N PLL, which generates a higher output frequency at 110.2 MHz. This paper focuses on proposing the design of an ultra-low-power, low-noise, low-complexity, and compact integer-N PLL with a MEMS-based reference oscillator as input. In particular, it introduces the design of an ultra-low-power, high-resolution, dead/blind zone-free phase-frequency detector (PFD), providing a smaller area and less design complexity. In addition, when combined with the used charge pump (CP), it offers smoother scalability between fabrication processes compared to traditional PFD designs. A charge transfer-based CP is used, offering lower power consumption, a smaller footprint, and a lower noise PLL design.

Section 2 briefly discusses the MEMS-based oscillator, showing the resonator and the sustaining amplifier circuit used in forming the reference oscillator for the PLL. The proposed PFD design and the different PLL building blocks are then discussed in Section 3. Section 4 covers the performance and the robustness of the proposed PFD circuit. Section 5 presents the experimental validation used to test the stand-alone PFD performance, as well as the measurement results of the overall system, and provides a comparison with state-of-the-art designs. Finally, Section 6 concludes the paper.

## 2. MEMS-Based Reference Oscillator

**Figure 2:**
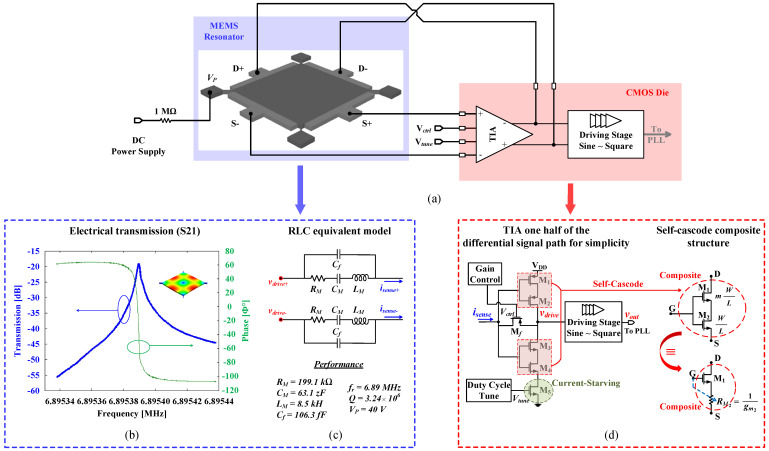
MEMS -based oscillator: (**a**) system block diagram; (**b**) measured MEMS electrical transmission (S21); (**c**) extracted RLC electrical equivalent linear model; (**d**) TIA circuit design.

Figure 2a shows a block diagram of the MEMS-based oscillator. The oscillator is built out of a differential bulk square MEMS resonator connected to a transimpedance amplifier (TIA) in a positive feedback loop to sustain oscillations.

### 2.1. MEMS Resonator

The oscillator built here is based on an electrostatic capacitive MEMS resonator. The resonator is explained in detail in [20]. A brief description is included here. The resonator is a bulk Lamé-mode wafer-level square resonator vacuum encapsulated at 10 mTorr. The resonator is fabricated using MEMS Integrated Design for Inertial Sensors (MIDIS) technology, a commercial process provided by Teledyne DALSA Semiconductor Inc., that features low-leakage and ultra-clean vacuum-level encapsulation. The resonator resonates at a 6.8953 MHz resonance frequency ($f_{r}$) with a *Q* factor of 3.24 × 10^6^ when polarized at a 40 V polarization voltage ($V_{P}$) and a frequency-quality factor (*f*-*Q*) product of 2.23 × 10^13^ Hz. The measured resonator frequency response at a $V_{P}$ of 40 V is shown in Figure 2b. Its RLC fitted linear electrical equivalent model, extracted at the same ($V_{P}$), is shown in Figure 2c [20], where $R_{M}$, $C_{M}$, $L_{M}$, and $C_{f}$ are the resonator’s motional resistance, motional capacitance, motional inductance, and feedthrough capacitance, respectively. Although the MEMS resonator is also measured at a maximum polarization voltage of 50 V, a lower voltage of 40 V is selected to reduce the requirements and complexity of the charge pump to be used to supply the polarization voltage.

### 2.2. TIA Design Methodology and Circuit Description

The TIA is presented in detail in [21]. A brief description is included here. The TIA utilizes a closed-loop negative shunt–shunt feedback configuration and a common-source (CS) topology in its design. The closed-loop configuration offers better noise performance and a better gain-bandwidth (GBW) trade-off relationship than the open-loop configuration [22]. The system input and output impedances are lowered by the loop gain due to the shunt–shunt feedback, which helps in increasing the TIA’s bandwidth. In addition, the input-referred noise of the amplifier is reduced by the square of the feedback resistor ($R_{f}^{2}$), which brings an extra benefit for low-phase-noise MEMS-based oscillators. The CS topology, in comparison to the common-gate and the common-drain topologies, helps in improving the dynamic range due to its higher output swing, which results in the lowering of the phase-noise floor [23]. In addition, it can be operated with a lower supply, resulting in less power consumption.

For an oscillator such as the one shown in Figure 2a, the total loop phase at the oscillation frequency ($f_{o}$) is zero. For a TIA phase shift (φ where |tanφ|≪Q), Equations (1) and (2) from [24] can be applied:

(1) $$f_{o}\approx f_{r}\left(1+\frac{tan\varphi }{2Q}\right),$$

where $f_{r}$ is the frequency of resonance. At $f_{o}$, a unity gain loop should be maintained; thus, the required TIA gain ($R_{TIA}$) can be expressed as follows:

(2) $$R_{TIA}\geq \frac{R_{M}}{\left|cos\varphi \right|}.$$

The TIA design methodology is based on the high *Q* of the used MEMS resonator. As illustrated by Equations (1) and (2), a tolerable amount of phase shift can be traded-off to save a considerable amount of power that would have been consumed for a larger bandwidth while not taking on the full benefit of the available high *Q*. Therefore, for a *Q* of $3.24\times 10^{6}$, a −80° phase shift only results in a 2 ppm change in $f_{o}$.

Thus, the TIA circuit shown in Figure 2d is based on three distinct features: (i) self-cascoding, (ii) current starving, and (iii) a long transistor channel length (*L*). The self-cascoding technique, which is typically used for low-voltage designs, reduces the channel-length modulation effect and offers a high-output swing and DC gain [25]. The current-starving technique is used in tuning the duty cycle by controlling the charging/discharging current flow, offering better noise performance [26,27], in addition to helping in consuming less power. Finally, increasing the transistor channel length results in reductions in the power consumption and the output phase noise.

Several factors affect the MEMS oscillator’s output phase noise, including the resonator *Q*, power handling, and frequency offset [28]. The higher the *Q* offered by the resonator, the lower the phase noise of the oscillator due to the improved noise filtering [24,29]. The higher the resonator’s power-handing capability, the lower the phase noise because of the increased sustainable signal amplitude in the oscillator. In addition, the phase noise is inversely proportional to the square of the frequency offset (Δf) from the resonant frequency ($f_{r}$); hence, the TIA noise is shaped by the oscillator’s feedback loop [30]. On the other hand, the phase noise is directly proportional to $R_{M}^{2}$ due to the higher TIA gain needed to compensate for the resonator losses.

The noise in the MEMS oscillator can be modeled by two main noise sources: (a) the resonator thermal noise, represented by $4k_{B}T/R_{M}$, where $k_{B}$ is the Boltzmann constant and *T* is the absolute temperature, and (b) the input-referred current noise ($\bar{i_{n}^{2}}$) of the TIA. For a high *Q*, where |tanφ|≪Q, and at a low (Δf), the oscillator phase noise is low and dominantly shaped by the high *Q* of the resonator, while the phase noise floor is proportional to $R_{M}^{2}$ and $\bar{i_{n}^{2}}$ [21]. Thus, the importance of minimizing the $\bar{i_{n}^{2}}$ is obvious. The negative-feedback shunt–shunt configuration helps to reduce the input-referred noise power spectral density of the amplifier by the square of the feedback resistance and helps to compensate for the directly proportional relationship of the noise floor with $R_{M}^{2}$. Neglecting the flicker noise, for the TIA design shown here, the thermal input-referred current noise can be expressed as

(3) $$i_{n}^{2}=4k_{B}T\left[\frac{\omega^{2}C_{in}^{2}\gamma }{g_{m1}+g_{m4}}+\frac{1}{R_{f}}\right],$$

where ω is the angular frequency; $C_{in}$ is the total input capacitance; γ is the transistor channel-length (*L*) noise coefficient; $g_{m1}$ and $g_{m4}$ are the transconductances of M_1_ and M_4_, respectively; $R_{f}$ is the shunt–shunt feedback resistor realized by transistor M_*f*_; and $k_{B}$ and *T* are the Boltzmann constant and the absolute temperature, respectively.

The TIA was developed using a TSMC 65 nm CMOS process instrument. The stand-alone TIA open-loop performance without the MEMS resonator was measured and studied thoroughly in [21]. A summary of the TIA open-loop performance parameters is shown in Table 1.

## 3. PLL Design

To meet the emerging reference oscillator demands of mobile electronics and future applications, the PLL was carefully designed to efficiently tackle power consumption and overall system performance. A block diagram of the integer-N PLL is shown in Figure 1. A type-II charge pump-based PLL was selected to achieve low power consumption [31], as detailed in this section. The PFD compares the phase and the frequency of the reference (REF) signal to the feedback (FB) signal and produces an error signal with the CP. This error signal is then filtered through a low-pass filter (LPF) to create a voltage-controlling signal ($V_{C}$) that controls the VCO and tunes its output oscillation frequency. With a divider in the feedback loop, the VCO output frequency is divided by the divider ratio (*N*), then compared with the REF. This forces the FB from the divider to have the same phase and frequency as the REF; hence, the PLL is in lock, which implies that at the VCO output frequency, the PLL output ($f_{OUT}$) is *N* times the REF signal’s frequency ($f_{REF}$).

To treat the PLL as a linear system, the PLL loop bandwidth should be much smaller than the REF frequency. A safe margin is to design the loop bandwidth at least 10 times smaller that the REF frequency to avoid the PFD sampling effect and approximate the PLL to a linear system [31]. In other words, the PFD-referred noise is low-pass-filtered by the loop cut-off frequency ($f_{c}$), while the VCO-referred noise is high-pass-filtered by $f_{c}$. Thus, the PFD-referred noise dominates at, low frequencies while the VCO-referred noise dominates at high frequencies. Therefore, the optimum choice of the PLL bandwidth is where the two noise sources intersect ($f_{c|optimum}$). The higher the PLL bandwidth ($BW>f_{c|optimum}$), the more PFD-referred noise allowed in the band and the lower the PLL bandwidth ($BW<f_{c|optimum}$), the more VCO-referred noise allowed in the band [15,31].

### 3.1. Loop Filter, VCO, and Divider

**Figure 3:**
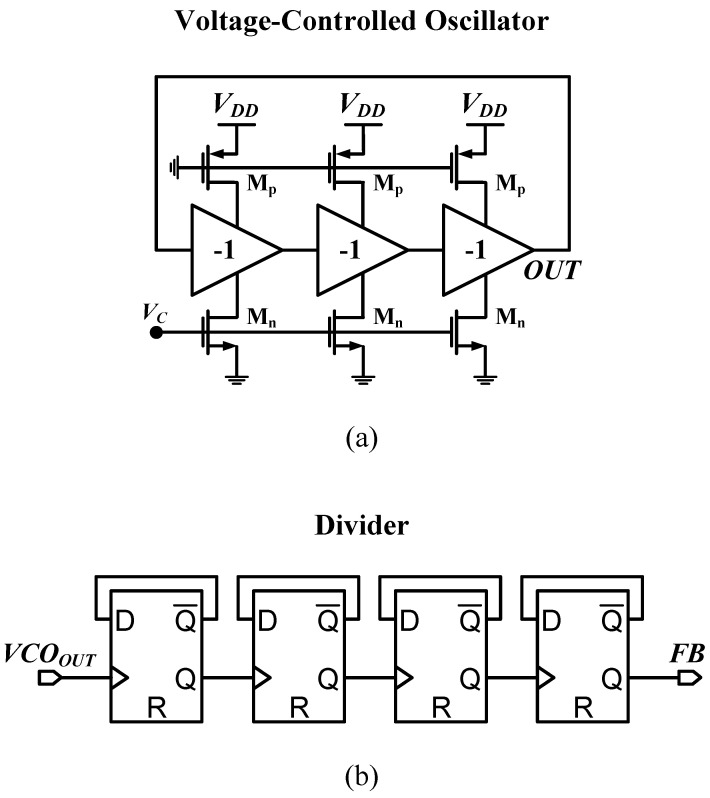
A simplified diagram of the PLL (**a**) ring-based VCO and (**b**) N=16 divider.

An on-chip first-order RC loop filter was designed to set the loop bandwidth around 50 kHz to minimize the overall noise of the voltage-controlled oscillator (VCO). The VCO is a current-starved, ring-based oscillator with a nominal frequency of 110.24 MHz designed with a long transistor channel length (*L*) for better noise performance and less power consumption [32,33]. A divider with a divide ratio (N) of 16 was designed based on low-power dynamic D flip-flops (DFFs) [34,35]. Figure 3 shows a simplified diagram of the VCO and the divider.

### 3.2. Charge Transfer-Based CP

**Figure 4:**
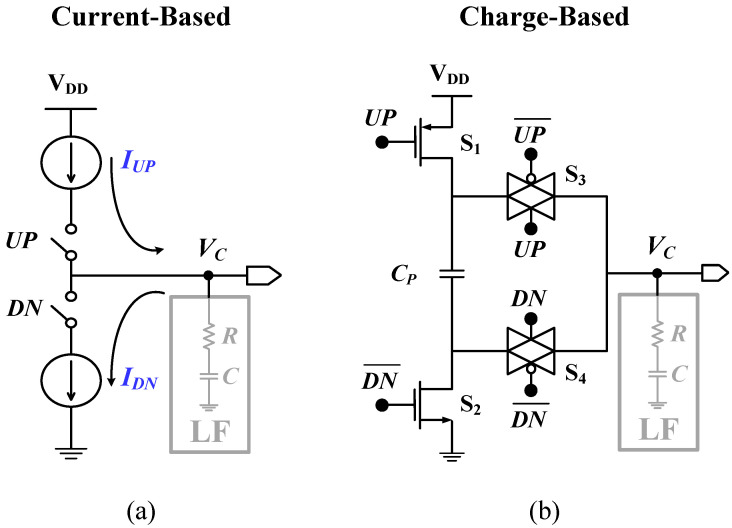
(**a**) Standard current-based and (**b**) charge transfer-based CPs.

The standard current-based CP shown in Figure 4a suffers from significant design challenges, including (a) wasted static power due to biasing and mirror current circuits that are always on, even in the phase-lock state; (b) the requirement for extra matching circuitry to match the charge-up current ($I_{UP}$) and charge-down current ($I_{DN}$); (c) a large active area; (d) a slow analog switching time because of the large transistor switches that take a longer time to switch on and off, adding more delay to the PLL control loop; (e) sensitivity to process variations; (f) limited headroom because of stacked transistors, resulting in restricted use in low-supply voltage applications; (g) current leakage through large switches, causing errors in the VCO control voltage ($V_{C}$) that affect the output frequency during locking; and (h) difficulty in scaling because of the large current mirrors and the large switches. Thus, a redesign is needed when transferring to an advanced small CMOS process technology node.

The charge transfer-based CP shown in Figure 4b, proposed by Schober and Choma [36], has a great advantage over the current-based CP, efficiently mitigating the aforementioned challenges. It does not suffer from device mismatch errors and can be operated at a low supply voltage. Most importantly, its fast-switching action allows for the use of a non-delayed PFD, which results in reduced reference spurs and low noise characteristics in the frequency spectrum of the PLL.

### 3.3. Phase-Frequency Detector

**Figure 5:**
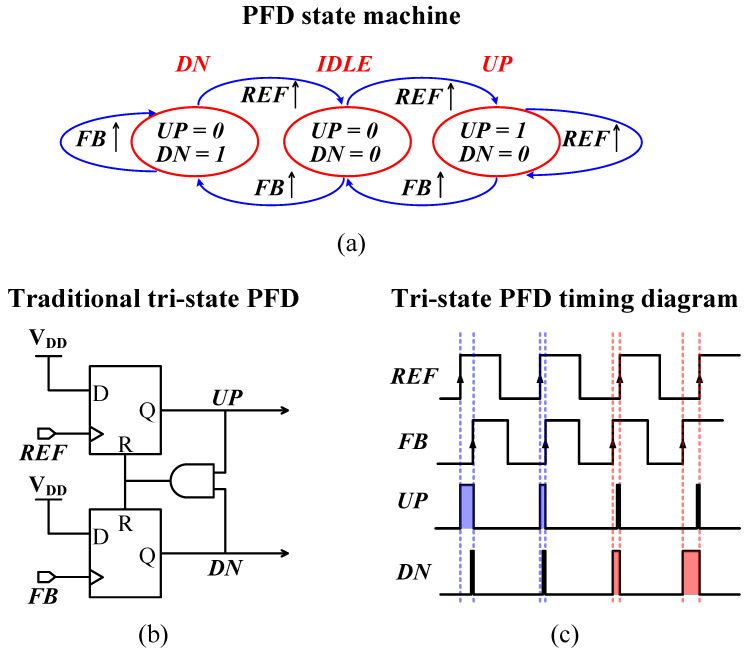
(**a**) A PFD state machine. (**b**) Traditional tri-state PFD block and (**c**) timing diagrams.

Phase-frequency detectors are used in a wide range of applications, including radar, interferometers, and system clocking. They are used in time-to-digital converters that have broad applications in time-of-flight (ToF) systems and all-digital PLLs [37], successive-approximation-register analog-to-digital converters [38], and time-mode signal processing. The PFD in a PLL plays a key role, along with the proper choice of CP topology. It operates in one of the three states shown in Figure 5a. The well-known tri-state PFD shown in Figure 5b is a sequential DFF based on a reset feedback-loop mechanism. In fact, this architecture suffers from several problems, including the mismatch between the fast propagation delay ($\tau_{p}$) of the DFFs and the slow analog switching times ($\tau_{s}$) of the CPs’ large switches, which causes the dead-zone issue. This is directly responsible for phase noise and spurious tones. Typically, this is mitigated by adding a delay circuit in the feedback reset path, which introduces an undesirable delay ($\tau_{d}$) to the PLL control loop that contributes significantly to the noise seen as jitter and acts as a source of instability [39]. Other techniques like dynamic-logic PFD (DL-PFD) have been proposed as candidates for high-frequency operation. They try to mitigate the dead-/blind- zone issues [40]. They operate on the principle of creating a delayed version of the input reference signal (REF) and the feedback signal (FB) after the divider in the PLL, with a delay larger than the reset time. The delayed feedback reset mechanism is not guaranteed to work as intended after fabrication and still needs additional calibration circuitry. A direct consequence of adding a delayed feedback path is the generation of an unwanted brief UP/DN 1/1 state to every cycle, as shown in Figure 5c, even during phase lock, which causes fluctuations in the CP and contributes to PLL jitter. This also increases both the power consumption and the delay-lock period of the PLL. To address the above issues the PFD proposed here (a) improves matching between the PFD logic ($\tau_{p}$) and the CP ($\tau_{s}$), (b) balances UP/DN signals for a given phase error, (c) causes no output glitches while in *idle* mode, (d) provides a wide frequency range of operation, and (e) is scalable across different CMOS fabrication processes.

## 4. Proposed PFD Design

The proposed PFD architecture is designed to integrate with a fast-switching, accurate charge transfer-based CP, where there is no need for the traditional delayed feedback reset mechanism. Rather, a PFD with the minimal possible delay is required, which, in turn, allows for a high resolution in phase-error correction, resulting in an extremely low level of added noise compared to other designs.

### 4.1. Architecture

**Figure 6:**
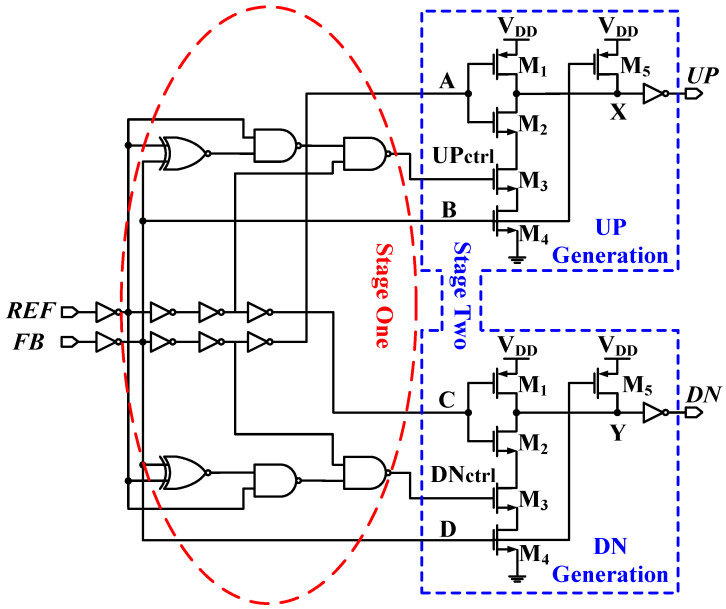
Circuit diagram of the proposed PFD.

Figure 6 shows the PFD design proposed here, consisting of two branches. One branch is responsible for generating the UP signal and resembles a phase-lead case—the REF signal leads the FB signal. The other branch is responsible for the DN signal generation for the case of a phase lag. Each branch consists of two stages. The first stage, shared between both branches, is used to generate a pulse width equal to the phase difference between the two input signals. The inverters in this stage are optimized and used to buffer the signal and guarantee a fast, steep rising edge ($\tau_{r}$). This pulse is then used as a control signal for the next stage. The second stage determines whether it is a phase-lead or phase-lag situation. Hence, it allows for only either an UP or a DN signal to be generated at any time. The inverter at the end buffers the output for better driving capability while delivering a positive pulse signal at node X (Y). The two stages operate together to replace the usage of DFFs, and the feedback reset path mechanism is commonly used in many PFD designs. Hence, no reset or dead/blind zones are present in this design. The system is enabled all the time and does not miss any of the edges at the inputs.

### 4.2. Circuit Design and Operation

As shown in Figure 6, the first stage consists of two NAND gates and one XNOR gate to measure the phase difference between the two input signals. Hence, an output control pulse with a width equal to the difference between the two input edges ($\Delta t_{in}$) appears at the output of the first stage. The second stage is implemented with five transistors per branch. Transistors M_1_–M_4_ act as a pull-down network that pulls down node X (Y) for a period of time equal to the input phase difference while blocking the other branch from generating any pulses. This guarantees that X and Y are never enabled at the same time. Transistor M_5_ is responsible for pulling up node X (Y) at the end of the created pulse. After the inverter, a positive pulse with a width equal to the phase difference between the REF and the FB input signals is created at one of the two branch outputs, depending on whether it is a phase-lead or phase-lag case.

### 4.3. Performance and Robustness

**Figure 7:**
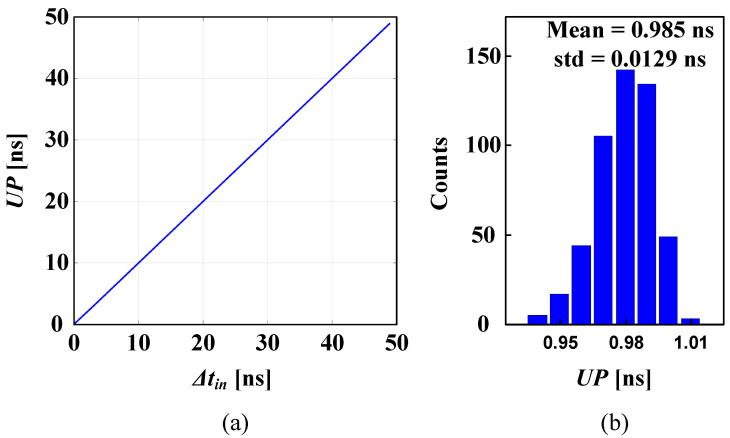
Proposed PFD: (**a**) transfer curve; (**b**) Monte Carlo histograms (N = 500) of the UP output at $\Delta t_{in}$ = 1 ns.

**Figure 8:**
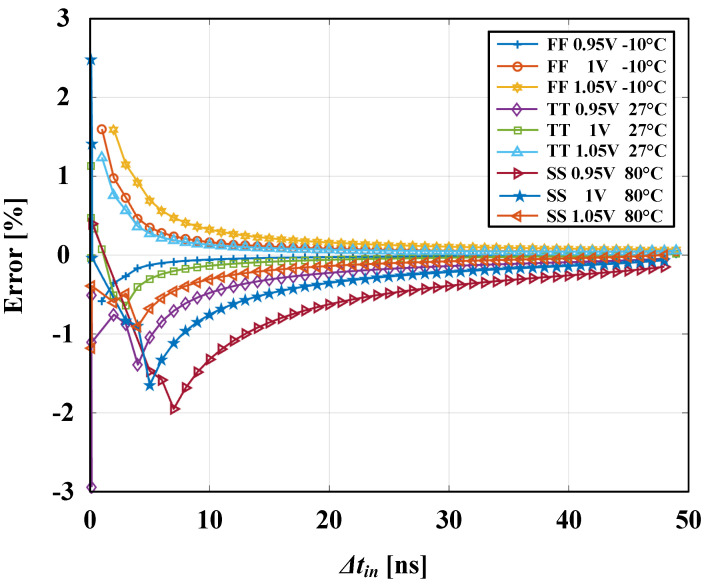
Error under PVT variations.

The proposed PFD was developed using a TSMC 65 nm CMOS process instrument. An open-loop simulation test was performed to check the PFD performance. First, a sweep test was carried out to check the resolution and the linearity under different input phase differences in the time domain based on $\Delta t_{in}$. The results shown here are based on a phase-lead case. Similar results were obtained for a phase-lag case. Figure 7a shows the output response of the PFD for different $\Delta t_{in}$ values when the REF signal leads the FB signal. The linear range starts from as low as 100 ps of resolution. To test the sensitivity of the proposed PFD circuit under transistor mismatches among the digital components, a Spectre post-layout simulation was carried out on the circuit shown in Figure 7b using a Monte Carlo analysis, with the number of runs set to N=500. Figure 7b shows a histogram of the PFD output for $\Delta t_{in}$ = 1 ns. It reports a mean of 0.985 ns and a standard deviation (std) of 0.0129 ns. The error is defined as

(4) $$Error=\frac{|PW_{out}-\Delta t_{in}|}{\Delta t_{in}}\times 100\%,$$

and calculated for different $\Delta t_{in}$ values, where $\Delta t_{in}$ is the input time difference and $PW_{out}$ is the corresponding pulse-width output. Figure 8 shows the post-layout simulated error across three process corners (FF, TT, and SS), a supply variation range of 1 V ± 0.05 V, and a temperature range of (−10 °C→+80 °C) to test the design robustness under process, voltage, and temperature (PVT) variations. The graph indicates a max error of ±3% in the pico-second range for all corners.

## 5. Experimental Validation

To validate the fabricated design, the PFD CMOS circuit was tested separately in an open-loop configuration while being loaded by the charge-based CP to validate its performance. Then, a test of the entire closed-loop system was performed after wire bonding the CMOS circuits to the MEMS resonator to realize the overall targeted system.

### 5.1. PFD Validation

**Figure 9:**
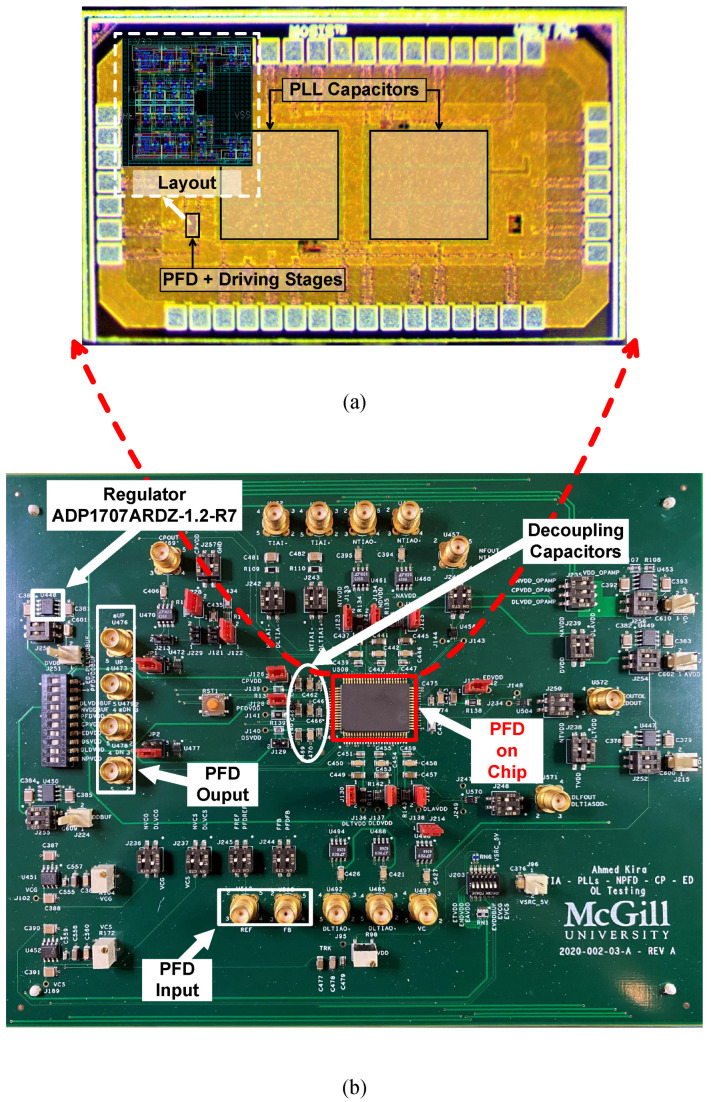
(**a**) Fabricated die micrograph; (**b**) photograph of the testing board used to test the PFD.

A measurement setup was built to test the PFD separately in an open-loop configuration without being connected in a closed-loop PLL. A copy of the designed PFD loaded with a charge-based CP was added separately on the die for the sake of carrying out this test without probing the closed-loop PLL. Figure 9a shows a micrograph of the fabricated die, highlighting the separate PFD block with its dedicated driving stage. The driving stage is used to drive the measuring equipment in the stand-alone PFD measurement. It is not a part of the fully integrated PFD in the closed-loop PLL. The total active PFD CMOS area is 92.29 μm^2^ (9.86 μm × 9.36 μm). The CMOS die was assembled in an 80-pin ceramic quad flat pack (CQFP) package, then mounted on a custom 4-layer printed circuit board (PCB), as shown in Figure 9b. The PCB includes SMA connectors for different input/output signals and a voltage regulator (Analog Devices ADP1707ARDZ-1.2-R7) that regulates the input supply from the DC supply source (Agilent E3646A). In addition, all power are decoupled with a network of decoupling capacitors (10 nF, 100 nF, 1 μF). The decoupling capacitors are connected in parallel and assembled in ascending order from the integrated circuit (IC) package lead. There are other components of the PCB shown in Figure 9b that are not related to this specific PFD test.

**Figure 10:**
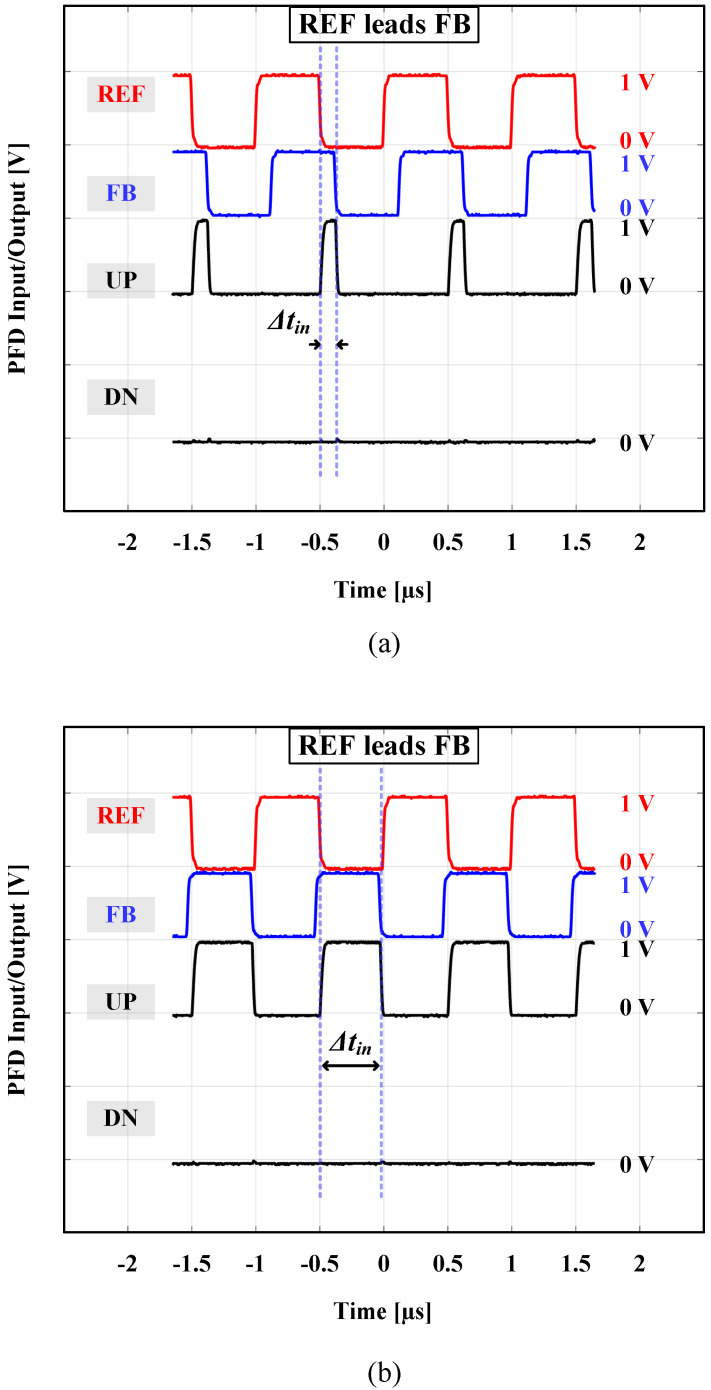
REF leads FB: (**a**) $\Delta t_{in}$ = 125 ns; (**b**) $\Delta t_{in}$ = 470 ns.

**Figure 11:**
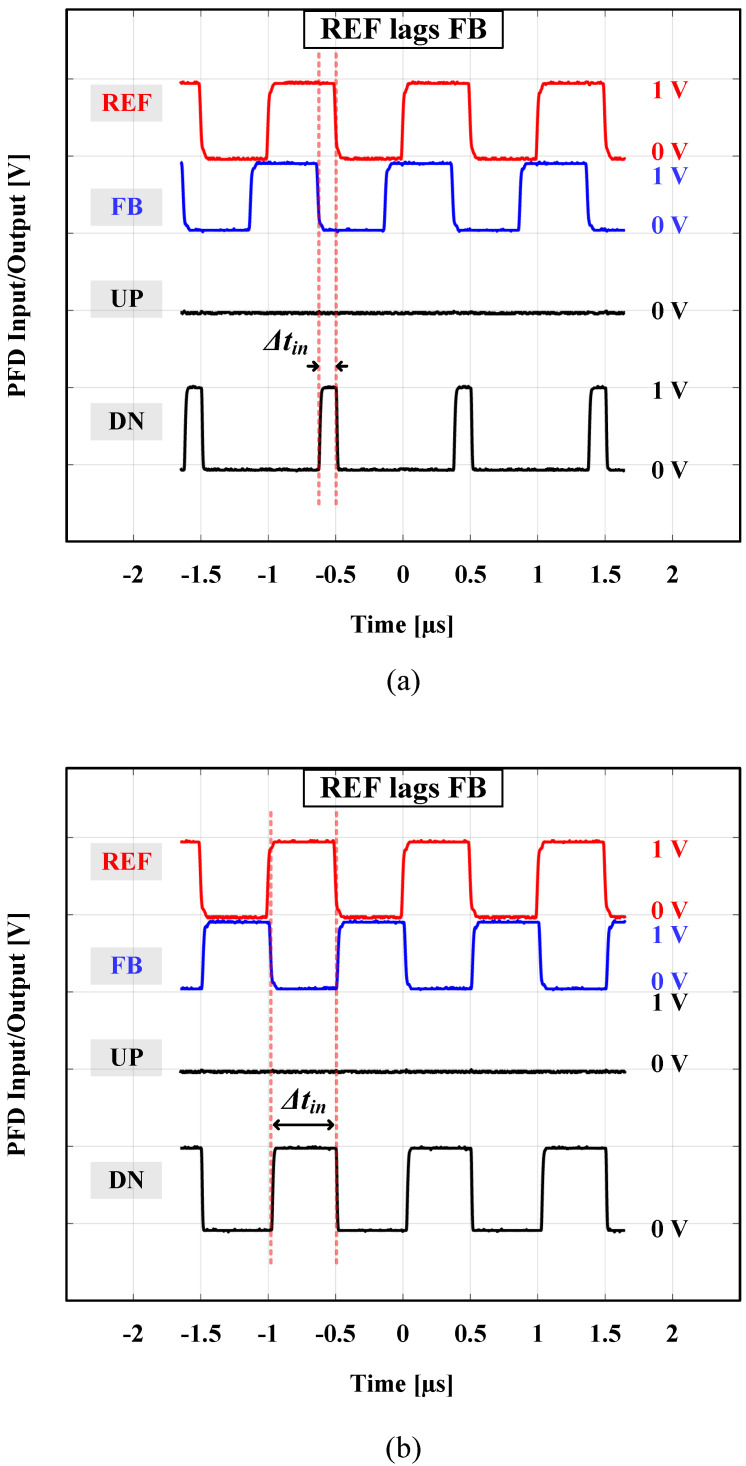
REF lags FB: (**a**) $\Delta t_{in}$ = −125 ns; and (**b**) $\Delta t_{in}$ = −470 ns.

Two input 1 V peak-to-peak synchronous square wave signals (REF and FB) were synthesized using two synchronized clock generators (Stanford Research Systems—Model CG635—2.05 GHz Synthesized Clock Generator) to feed the PFD block under test. One CG635 clock generator was used to generate the REF signal, and the other one was used to generate the FB signal with a controllable $\Delta t_{in}$ time difference (phase shift) out of the REF signal. An oscilloscope (Keysight/Agilent MSO-X 92504A) was used to display the PFD input, REF, and FB signals. Another oscilloscope (Tektronix MSO71254C) was then used to monitor the 4 PFD output signals: UP, $\bar{UP}$, DN, and $\bar{DN}$. Figure 10a shows the measurement of the PFD output at a 1 MHz operating frequency when REF leads FB by π/4 ($\Delta t_{in}$ = 125 ns), and Figure 10b shows the case of REF leading FB by almost π ($\Delta t_{in}$ = 470 ns). It is obvious that in both cases, there are not any generated pulses or glitches in the DN output. Figure 11a depicts the case when REF lags FB by −π/4 ($\Delta t_{in}$ = −125 ns), and Figure 11b shows the case when REF lags FB by almost −π ($\Delta t_{in}$ = −470 ns). Similarly, no pulses or glitches are generated in the UP output.

**Figure 12:**
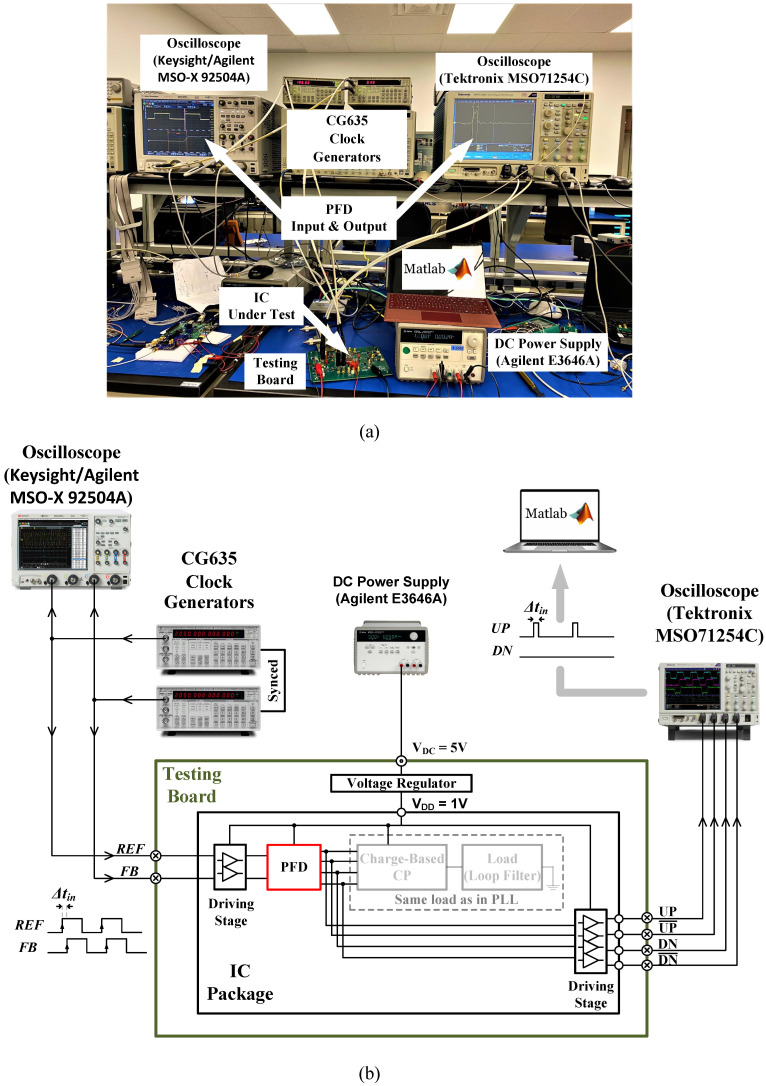
Stand-alone PFD validation: (**a**) picture of the actual setup; (**b**) setup block diagram.

**Figure 13:**
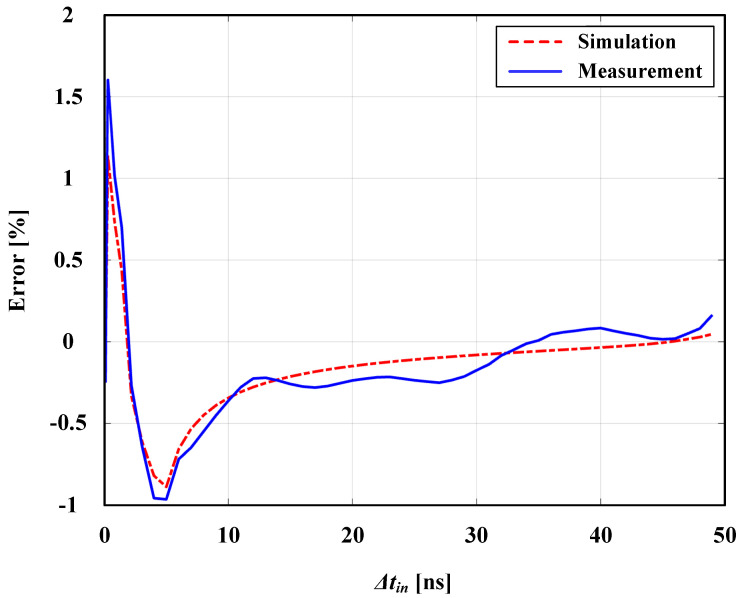
Measured PFD error compared to simulation.

The actual measurement setup and its block diagram are depicted in Figure 12. Figure 13 shows the measured error of the PFD. It reports a maximum absolute measured error of around 1.6% in the pico-second range of $\Delta t_{in}$ and ≤1% in the nano-second range. The result is in agreement with the simulation. The PFD frequency-normalized power consumption ($P_{n}$) is 0.106 pW/Hz at a 1 V supply voltage. The performance of the proposed design is summarized in Table 2, along with other works reported in the literature. Compared to other designs, this work reports the lowest $P_{n}$ and the lowest percentage of error in the PFD output, with a competitive maximum operating frequency ($f_{max}$) that covers the intended application range of operation.

### 5.2. Prototype and Overall Closed-Loop System Validation

**Figure 14:**
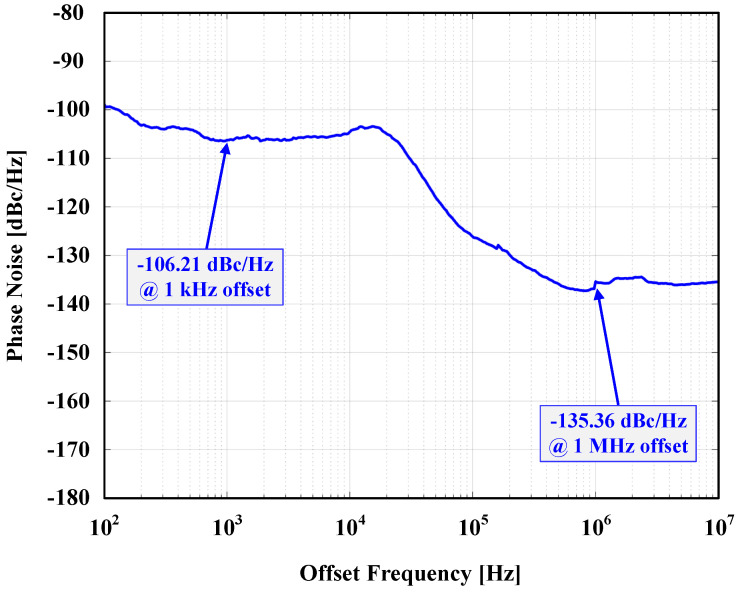
Measured phase noise at a 110.2 MHz output frequency.

**Figure 15:**
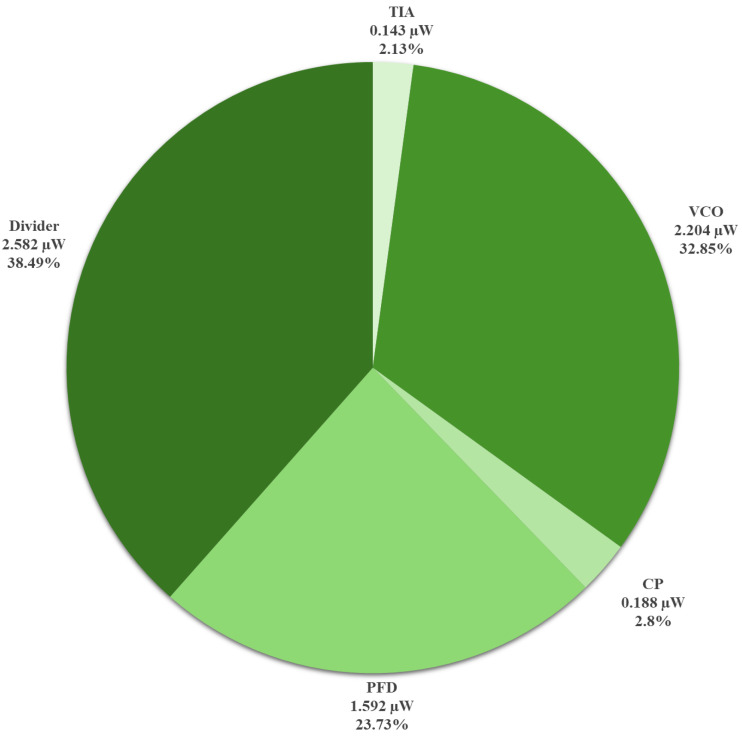
Breakdown of the power consumption of the system at a 110.2 MHz output frequency.

**Figure 16:**
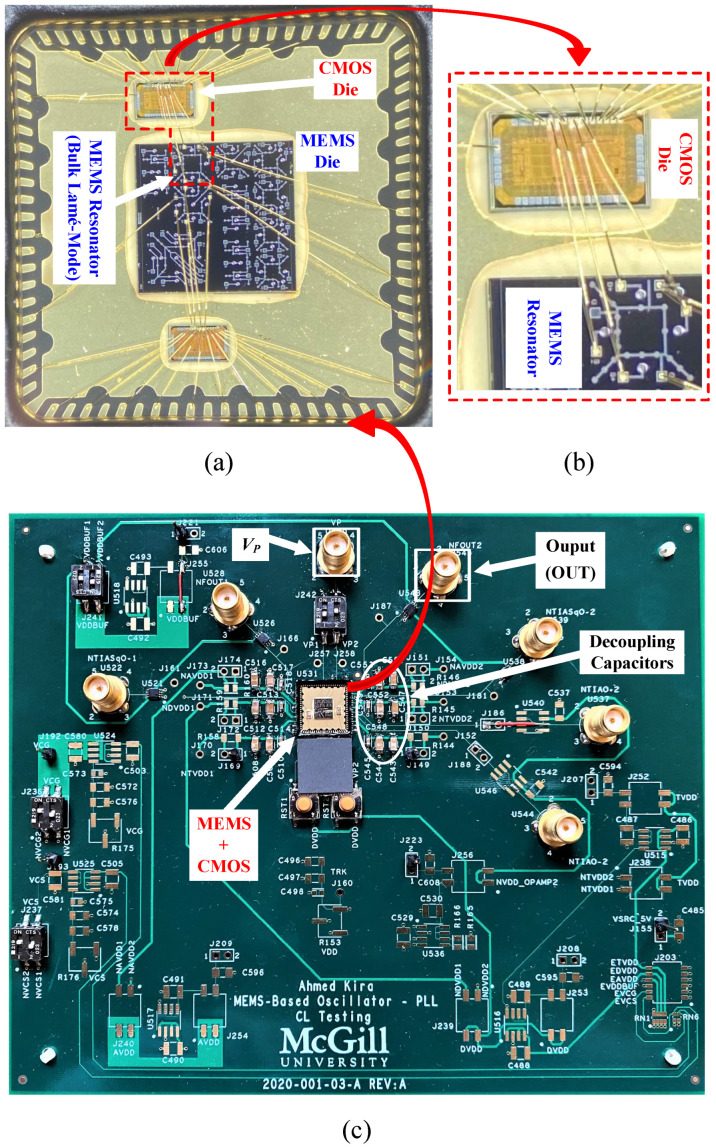
(**a**) Picture of the wire-bonded dies in the package. (**b**) Zoomed-in view of the MEMS device wire-bonded to the CMOS die, forming the system. (**c**) Photograph of the testing board used to test the system.

The loop was then closed, connecting the MEMS resonator to the TIA, forming the MEMS oscillator to test the overall system, including the PLL with MEMS-based reference oscillator as input. The MEMS and CMOS dies are wire-bonded to each other. An 80-pin quad flat no-lead (QFN) package was used for packaging. QFN packaging was chosen over quad flat pack (QFP) because of the lower loading capacitance of its pads [44,45]. A different custom PCB was designed for this test. A network of decoupling capacitors (10 nF, 100 nF, and 1 μF) was connected in parallel and assembled in an ascending order from the lead of the IC package to decouple all the power nets. The MEMS $V_{P}$ was supplied by a DC power supply source (BK PRECISION 9110) with a 1 MΩ resistor connected in series between the resonator and the supply generator. The 1 MΩ series resistor helped in limiting the maximum supply current to the polarization terminal in the case of pull-in between the MEMS device electrodes. The CMOS circuits were supplied with a 1 V supply voltage from another DC supply source (Agilent E3646A). The MEMS ($V_{P}$) was gradually raised up to observe MEMS behavior, and a stable output was reached at $V_{P}=40$ V. Figure 14 shows the phase noise of the output after the PLL. It reports phase noises of −106.21 dBc/Hz and −135.36 dBc/Hz at 1 kHz and 1 MHz offsets, respectively. At a 1 V supply, the total power consumption of the PLL is 6.566 μW, and the total power consumption of the system (P_DC_), the TIA, and the PLL is 6.709 μW. Figure 15 shows the breakdown of the system power consumption at a 110.2 MHz output frequency. Figure 16 shows the wire-bonded dies in the mounted package and the testing PCB.

To be able to fairly compare the performance of different oscillators, a well-known figure of merit in [46,47] and based on the oscillator noise and power consumption was utilized in this work. It normalizes the oscillator phase noise at a specific offset frequency (Δf) to the oscillator’s frequency of oscillation ($f_{o}$) and to the DC power consumption [48]. The FoM is expressed as follows:

(5) $$FoM=L\left(\Delta f\right)-20\log\left(\frac{f_{o}}{\Delta f}\right)+10\log\left(\frac{P_{DC}}{1\;\mathrm{mW}}\right),$$

where L(Δf) is the oscillator output phase noise at a specific offset frequency (Δf), $f_{o}$ is the oscillator’s frequency of oscillation, and $P_{DC}$ is the oscillator’s DC power consumption in milliwatts.

FoM is used in Table 3 to compare the results of this work with those of previously published state-of-the-art methods. The system demonstrated in this work reports a competitive FoM compared to other works reported in the literature.

## 6. Conclusions

This paper presents the implementation of a 110.2 MHz ultra-low-power PLL using a MEMS-based input reference oscillator as a replacement for an external quartz crystal-based reference. A TIA based on the self-cascoding technique was integrated with a very high-*Q* bulk Lamé-mode MEMS resonator to build an oscillator with competitive performance.

An ultra-low-power, dead/blind zone-free, glitch-free, high-resolution PFD was successfully implemented. The PFD structure consists of two branches with two stages that replace the usage of any feedback reset mechanism and allow only for the generation of either a UP or DN signal at a time. A stand-alone experimental validation was performed for the PFD separately. The results report a resolution of 100 ps with less than ±1.6% error and a frequency-normalized power consumption ($P_{n}$) of 0.106 pW/Hz. They show a compact, accurate, and efficient PFD with competitive performance compared to other state-of-the-art methods.

The whole system was fabricated using a TSMC 65 nm CMOS process and experimentally validated to test the overall performance. It occupies a total active CMOS area of 0.1095 mm^2^ and consumes a total power of 6.709 μW at a 1 V supply. The system phase noises re −106.21 dBc/Hz and −135.36 dBc/Hz at 1 kHz and 1 MHz offsets, respectively. The proposed design can be easily transferred and adapted for new advanced CMOS technologies with lower supply conditions.

## Figures and Tables

**Table 1:** TIA open-loop performance summary.

Parameter	Measured Values
Process (nm)	65
Supply (V)	1
Gain (dBΩ)	107.9→118.1
Duty Cycle (%)	23.25→79.03
Power Consumption (nW)	143
Active CMOS Area (μm^2^)	150.29

**Table 2:** PFD performance summary and comparison.

Ref. No.	[41]	[42]	[43]	This Work
Process (nm)	90	65	180	**65**
Supply (V)	1.2	1.2	1.8	**1**
$P_{n}$ (pW/Hz)	-	0.62	0.132	**0.106**
$f_{max}$ (GHz)	6	0.1	2.5	**2.5**
Dead zone (ns)	Near 0 *	-	Free	**Free**
Error (%≤)	±15	±12 ^•^	-	**±1.6**

* The authors did not report how close it was to 0. ^•^ Extracted from the reported results.

**Table 3:** Summary of system performance and comparison with the state of the art.

Ref. No.	[47] ^•^	[49]	[50] ^•^	[51]	[52]	[53]	This Work
Process (nm)	180	65	180	40	65	65	**65**
PLL Architecture Type	Analog Fractional-N	Digital Fractional-N	Analog Fractional-N	Analog Integer-N	Analog Integer-N	Digital Integer-N	**Analog Integer-N**
Supply Voltage (V)	-	0.85 ^⊗^	1.5 ^+^	1	1	0.8 *	**1**
Reference Frequency (MHz)	27.19	10	5	250 *	10	2 *	**6.89**
Output Frequency (MHz)	75.01	2466	100 *	25,000 *	1000	240 *	**110.24**
P_DC_ (μW)	7800	265	3000 ^$^	1080	320	63.5	**6.709**
Power Efficiency (μW/MHz)	103.99	0.1075	30	0.0432	0.32	0.26	**0.0609**
Core Active CMOS Area (mm^2^)	5 ”	0.25	0.36	0.0048	0.315	0.016	**0.1095**
PN|_1kHz_ (dBc/Hz)	−108	−68	−84.2907	−98 ^!^	−64 ^!^	−44.34	**−106.21**
PN|_1MHz_ (dBc/Hz)	−133.15	−105	−134.1	−121.1	−73	−76.48	**−135.36**
FoM|_1kHz_ (dB)	−196.58	−190.07	−179.52	−246.29	−179.05	−139.97	**−185.32**

^•^ The used input reference: MEMS-based input reference oscillator. ^⊗^ The DCO works at a 0.45 V supply, while the rest of the DPLL is supplied with a 0.85 V supply. ^+^ Internal supply voltage of the core CMOS circuits. * Value selected by the authors of the paper from a tunable range within which they measured the reported data. ^$^ Estimated for an internal, core CMOS and PLL circuit blocks. ” The whole ASIC CMOS chip size; core areas of different circuits are not mentioned. ^!^ Deduced from the reported data.

## Data Availability

Data are contained within the article.
